# A randomized study comparing regular care with a nurse‐led clinic based on tight disease activity control and person‐centred care in patients with rheumatoid arthritis with moderate/high disease activity: A 6‐month evaluation

**DOI:** 10.1002/msc.1403

**Published:** 2019-06-20

**Authors:** Ulrika Bergsten, Katarina Almehed, Amir Baigi, Lennart T.H. Jacobsson

**Affiliations:** ^1^ Research and Development Department at Region of Halland Halmstad Sweden; ^2^ Rheumatology Department Sahlgrenska University Hospital Goteborg Sweden; ^3^ Department of Rheumatology and Inflammation Research University of Gothenburg Goteborg Sweden

**Keywords:** nursing, rheumatoid arthritis, self‐management

## Abstract

**Introduction:**

A recent survey showed that 27% of rheumatoid arthritis (RA) patients had inadequately controlled disease activity. Hence, there is a need for new strategies aiming at improving patient outcomes. The aim of the present study was to evaluate the effect of a nurse‐led clinic with frequent visits, treat‐to‐target and person‐centred care of patients with established RA and moderate‐to‐high disease activity compared with patients receiving regular care.

**Methods:**

The study was a randomized, controlled trial over 26 weeks, with a nonrandomized extension to week 50. Patients were randomized to an intervention group (IG; nurse‐led clinic) based on person‐centred care, frequent visits and “treat to target”, or to a control group (CG) which visited the clinic according to care as usual. The primary outcome was the difference in the DAS28 change between the IG and the CG groups.

**Results:**

A total of 332 patients were screened for eligibility, of which 70 were randomly assigned to either the IG (*n* = 36) or the CG (*n* = 34) group. The primary outcome was not met, although patients in the IG group tended to improve more than those in the CG group (difference: 0.43 (95% confidence interval [CI] –0.27, 1.13). In both the IG and CG groups, delta‐DAS28 improved significantly. The European League Against Rheumatology moderate or good response was achieved by 76% (95% CI 58, 89) in the IG and 49% (95% CI 32, 65) in the CG group.

**Conclusions:**

Disease activity tended to improve more with the nurse‐led intervention compared with regular care, although the difference was not significant, probably partly due to the lack of statistical power.

## 1 INTRODUCTION

Rheumatoid arthritis (RA) is a chronic autoimmune disease, characterized by persistent inflammation and, if insufficiently treated, progressive destruction of the joints (Klareskog, Catrina, & Paget, 2009). Other adverse outcomes are pain, fatigue, disability and the loss of ability to participate in valued activities (Wikström, Book, & Jacobsson, 2006; Wikström, Jacobsson, & Arvidsson, 2005).

Since the 1990s, numerous biological pharmacological therapies have been found to be effective for RA and introduced into clinical practice, often to be used in combination with older, conventional synthetic disease‐modifying anti‐rheumatic drugs (csDMARDs; mainly methotrexate). To support a rational use of these, often expensive, therapies, guidelines have been published, both at a European level (Nam et al., 2017; Smolen et al., 2017) and on a national level in Sweden (National Board of Health and Welfare, 2012; Svensk reumatologisk förening, 2018), the latter updated yearly since 2007. The key factors emphasized by all guidelines are to: (a) follow the described algorithms adjusted to local practice with sequential use of immune modulating drugs; (b) start early pharmacological treatment to reduce/abolish the inflammation and destruction of the joints; (c) achieve the lowest possible disease activity (“treat to target”) with frequent visits (“tight control”); and (d) involve the patient in their care through shared decision‐making (Nam et al., 2017; Smolen et al., 2017).

Despite these guidelines, 35% of patients in the Swedish Rheumatology Quality Register (SRQ) had a Disease Activity Score in 28 joints (DAS28) indicating moderate or high disease activity in 2017, and in a recent survey in five large European countries 27% of RA patients had inadequately controlled disease activity, defined as not having a DAS28 below 3.2. There is thus a need for new strategies to improve patient outcomes. One such strategy could be to focus on the individual patient's needs and own goal setting, and to address their fear of the disease and medication (Gossec et al., 2018; Nam et al., 2017). Nurse‐led clinics could be one way to strengthen the position of the patient in the decision‐making, and the European League Against Rheumatology (EULAR) emphasizes the importance of studying the effect of such strategies, and has developed guidelines for the role of the nurse in the management of RA (van Eijk‐Hustings et al., 2012).

Several publications on nurse‐led clinics in rheumatology have been published over the last decade(s), mostly performed in patients with low disease activity or in remission (Arvidsson et al., 2006; Bala et al., 2012; Hill, Thorpe, & Bird, 2003; Larsson, Fridlund, Arvidsson, Teleman, & Bergman, 2014; Ndosi et al., 2014; Ndosi, Vinall, Hale, Bird, & Hill, 2011; Tijhuis, Zwinderman, Hazes, Breedveld, & Vlieland, 2003). Although there is a relative lack of randomized clinical trials investigating the effect of nurse‐led clinics in patients with moderate or high disease activity, there is support for the notion that the nurse‐led clinics can: improve the patient's function; increase the patient's knowledge about the disease (Hill et al., 2003; Tijhuis et al., 2003); improve self‐efficacy (Primdahl, Wagner, Holst, & Hørslev‐Petersen, 2012); and add value for the patient, in terms of increased security, continuity and a positive feeling of being seen as a person (Arvidsson et al., 2006; Bala et al., 2012; Larsson, Bergman, Fridlund, & Arvidsson, 2012). This led us to hypothesize that a nurse‐led clinic built on principles of person centred care and stringent follow‐up, with tight control and treat‐to‐target strategy, would be more effective than care as usual in patients with RA with moderate to high disease activity.

The aim of the present randomized, controlled study was thus to compare the effect of a nurse‐led clinic (including person‐centred care and frequent visits aiming at disease activity remission) with that of patients seen in regular care, in patients with established RA with moderate to high disease activity.

## 2 METHODS

### 2.1 Trial design and participants

**Figure 1:**
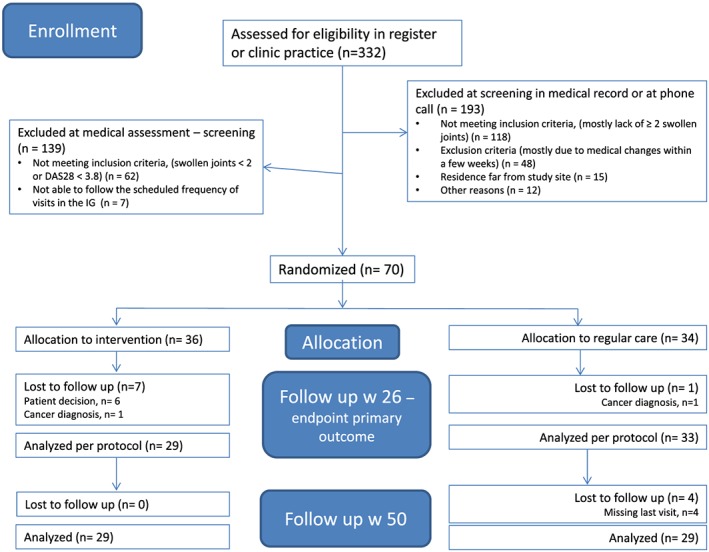
Flow chart for enrolment. DAS28: Disease Activity Score in 28 joints; IG: intervention group [Colour figure can be viewed at http://wileyonlinelibrary.com]

The study was a randomized, controlled, assessor‐blinded study comparing the effect of a nurse‐led clinic (including person‐centred care and treatment according to tight control and a treat‐to‐target strategy) with regular care in patients with established RA with moderate to high disease activity (Figure 1). Eligible participants were adult patients (aged 18–80 years) with RA of over 2 years’ duration, moderate to high disease activity (DAS28 >3.8, two or more swollen joints) and with stable medical treatment for >8 weeks. This level of disease activity was set to enable the inclusion of patients with considerable disease activity, who we hypothesized would benefit from components of the intervention. Exclusion criteria were a diagnosis of any other inflammatory arthritis; history of chronic infection; concurrent malignancy or history of malignancy; or current or recent history of any other severe, progressive or uncontrolled comorbidity. In order to enable escalation of pharmacological therapy, patients who had discontinued all of the following medications (either due to lack of effect or adverse effects) were also excluded from the study: methotrexate, sulfasalazine, ≥2 tumour necrosis factor inhibitors, abatacept, rituximab and tocilizumab.

Patients were recruited either at a regular care visit at the rheumatology clinic or through screening of the previous year's recorded visits in the SRQ. If the patient at a regular visit had a moderate to high disease activity (DAS28 >3.8), the rheumatologist informed the patient about the study. In addition, the SRQ and the clinical records were used to screen for patients with RA and moderate to high disease activity. Patients fulfilling the inclusion and exclusion criteria at their last recorded visit were contacted by telephone or letter, and informed about the study. Patients who were interested in participating were scheduled for a screening visit, and if the patient consented to participate, randomization was performed at this visit, which was also the baseline visit for both study arms.

The study was approved by the regional ethics committee at Gothenburg (Dnr 855–13).

### 2.2 Nurse‐led clinic (intervention group)

The focus of the patient visit in the intervention group (IG) (i.e., the nurse‐led clinic) was on person‐centred care and tight control, with a treat‐to‐target strategy with regard to disease activity. The four nurses involved in the intervention received 2 days’ training in the philosophy and delivery of person‐centred care, the principles of treat to target and the study design. Furthermore, the study nurses had undergone training in joint examination, and been certified, at the clinic before the study began. Person‐centred care is defined as a partnership between patients and healthcare professional. The starting point is to listen to the patient's narrative, which, along with other examinations, forms the basis for a health plan (Ekman et al., 2011). The partnership includes the shared decision‐making that is highlighted in the EULAR guidelines (Nam et al., 2017).

**Figure 2:**
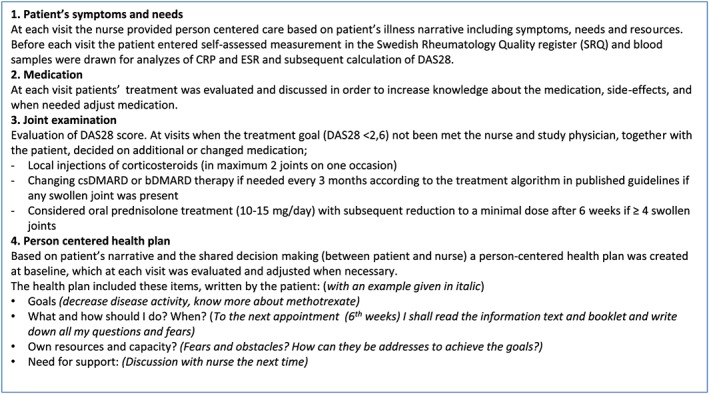
Description of the contents of the visits in the intervention group. bDMARD: biologic disease‐modifying anti‐rheumatic drug; CRP: c‐reactive protein; csDMARD: conventional synthetic disease‐modifying anti‐rheumatic drug; DAS28: Disease Activity Score in 28 joints; ESR: erythrocyte sedimentation rate [Colour figure can be viewed at http://wileyonlinelibrary.com]

At the study visit 2 weeks later, an individual health plan was decided on and documented by the patient and the study nurse (Figure 2). The health plan contained the overall aims, with regard to targets for disease activity and participation in the patient's highest prioritized activity. The health plan also included specific tools on how to achieve these goals, such as gaining more knowledge about the disease or treatment, care from a physiotherapist/occupational therapist/counsellor for consultations, or changing pharmacological treatment, with specified follow‐up routines. Every 6th week thereafter, the DAS28 was assessed at a study‐nurse visit, and the nurse and patient together re‐evaluated the health plan. If disease remission (DAS28 <2.6) had not been reached *and* there was residual inflammatory activity with ≥1 swollen joint at examination, a change of pharmacological treatment was considered through consultation with a study physician. Pharmacological therapy was changed according to the yearly updated Swedish guidelines (Svensk reumatologisk förening, 2018), which are in accordance with the EULAR guidelines for the treatment of RA (Smolen et al., 2017).

Patients who had attained a low disease activity (DAS28 ≤3.2) at week 26 in the IG were further followed in a similar fashion to those in the control group (CG), with only one scheduled additional follow‐up at week 50 during the extended follow‐up (weeks 26–50). Patients with a DAS28 >3.2 at week 26 continued to visit the nurse‐led clinic every 6th week until the end of the extended follow‐up (weeks 26–50).

### 2.3 Regular care group (CG)

The patients in the CG visited a nurse at the clinic, who was blinded to randomization, for an independent joint assessment at baseline, week 26 and week 50. After randomization, the patients in the CG were offered a telephone appointment with their regular physician, in order to discuss their disease activity and whether a physical appointment, and potentially a change in therapy, should be made. This option was used by 23/34 (70%), and in 20 of these this contact resulted in either increased dosing or a change in DMARD therapy. All patients in the CG were then followed by their treating physician according to regular care, with follow‐up visits decided either at this telephone appointment or according to previous plans. In regular care, the patients usually visited the clinic every 6–12 months (according to data from the SRQ). As part of regular care, patients also had the possibility of making appointments with the physician in the event of flares. If the csDMARD or biologic DMARD (bDMARD) therapy was changed at a regular visit, the patient usually was given an appointment with a rheumatology nurse within 2 weeks for information and follow‐up, as part of the routine in regular care.

### 2.4 Outcomes

The primary outcome was the difference in the DAS28 change between the IG and the CG groups at week 26. DAS28 is an index based on the number of tender and swollen joints, patients’ global health assessment and the erythrocyte sedimentation rate (14). DAS28 was assessed at baseline, week 26 (primary endpoint) and at week 50, with swollen and tender joint counts assessed by an assessor blinded to randomization.

Secondary outcomes at week 26 were the difference between the IG and the CG groups in: (a) the proportions with minimal clinical important improvement in DAS28 (>0.6); (b) the proportions achieving low disease activity (DAS28 <3.2); (c) the proportions achieving a EULAR moderate or good response (Prevoo et al., 1995); (d) the Health Assessment Questionnaire score, measuring daily function (Ekdahl, Eberhardt, Andersson, & Svensson, 1988); (e) the RA impact of disease (RAID) score, measuring the impact of RA from the patient's perspective (Gossec et al., 2011); (f) Patient Acceptable Symptom State (PASS) score (Tubach et al., 2005); (g) the Beliefs about Medicines Questionnaire (BMQ) responses, measuring patients’ attitude to medication split in two domains (BMQ‐necessity, BMQ‐concerns) (Horne, Weinman, & Hankins, 1999); and (h) the EuroQol‐5D (EQ‐5D) score (EuroQol Group, 1990).

#### 2.4.5 Sample size calculations

Based on historical estimates of patients identified in the SRQ at the Sahlgrenska University Hospital in Gothenburg from January 2011 to December 2012, 19% of patients with RA had a disease activity, as defined by inclusion and exclusion criteria (*n* = 401).

According to these historical data, at the first follow‐up with regular care, the reduction in the DAS28 was 1.43 (standard deviation [SD] 1.50). We assumed that we would have a larger improvement in the IG group, of 2.0 (SD: 1.50). Based on this assumption, a sample size of 50 evaluable patients per study arm (total sample size 100) provided the trial with 80% power to detect a significant difference (*P* < 0.05) between the two treatment arms at 26 weeks. In order to allow for at least a 10% dropout rate, we decided to include 60 patients in each study arm.

For several reasons, including competing studies and an improvement in the standard of regular care during the recruitment period, we encountered increasing problems with recruiting patients for this single‐centre study. Recruitment to the study therefore had to be terminated before the planned number of patients had been included.

#### 2.4.6 Randomization

Patients fulfilling the inclusion and exclusion criteria and who had signed informed consent were randomized (1:1) into one of the following two groups: the nurse‐led clinic group (IG) or the regular care group (CG). Randomization was performed using minimization incorporating random elements. We used a computer program for the randomization process. Minimization was employed to ensure that treatment arms were balanced for DAS28 at baseline, age and sex. Study nurses/physicians and patients were all aware of the allocation. Follow‐up assessments (joint assessment at weeks 26 and 50) were performed by a research nurse blinded to randomization.

### 2.7 Statistical methods

Baseline characteristics are given as means (standard deviation [SD]) and frequencies. The results were analysed both by per‐protocol (PP) and intent‐to‐treat (ITT). As patients in the CG were not assessed at intermediate time points from baseline to week 26, it was considered most appropriate, as stated in the protocol, primarily to analyse the study population PP, although ITT analyses were also performed and are presented here. In the ITT analyses, the last observation carried forward (LOCF) method was used. In the IG, LOCF was based on the mean of all assessed values up to the date of exclusion, whereas in the CG baseline values were carried forward from the baseline visit (owing to the lack of assessments between baseline and week 26). Groups were compared using a t‐test for independent samples, and 95% confidence intervals (CIs) were calculated. The differences between groups for categorical variables were calculated by use of the chi‐square test. The level of significance was set at 0.05.

## 3 RESULTS

**Figure 3:**
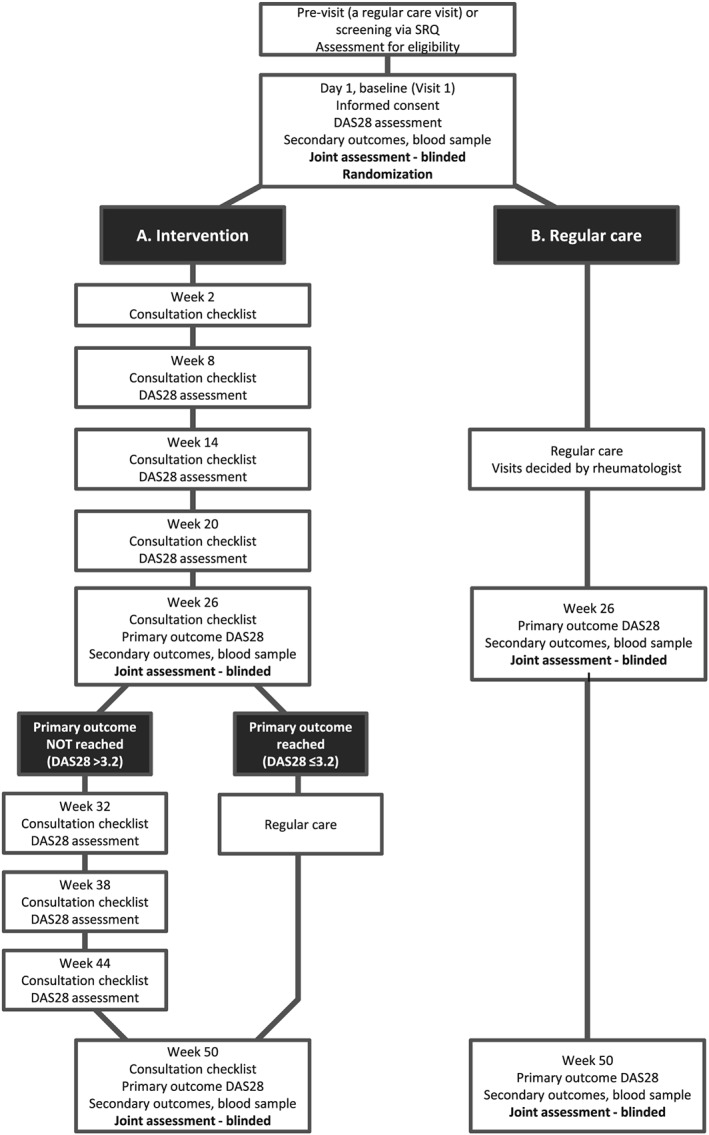
Trial profile. DAS28: Disease Activity Score in 28 joints; SRQ: Swedish Rheumatology Quality Register

A total of 332 patients were screened for eligibility and, of these, 70 (21.1%) patients were randomly assigned to either the IG (*n* = 36) or the CG (*n* = 34) (Figure 3).

In the IG, 81% (*n* = 29/36), and in the CG, 97% (*n* = 33/34) of patients completed treatment and follow‐up through to week 26. Baseline characteristics were similar across treatment arms for the stratification variables age, sex and DAS28. Smaller differences were noted for other variables, with higher frequencies in the CG of seropositivity for rheumatoid factor or anti‐citrullinated protein antibodies, most comorbidities, ongoing csDMARD and bDMARD treatment, and mean value for RAID, whereas in the IG combination therapy with csDMARDs was slightly more common (Table 1).

**Table 1:** Baseline characteristics of the study population, overall and stratified by study groups, given as mean (standard deviation) or *n* (%)

Parameter	Intervention group (*n* = 36)	Control group (*n* = 34)	Total (*N* = 70)
Age (years)	60.3 (15.9)	62.4 (12.2)	61.3 (14.2)
No. of women	31 (86%)	28 (82%)	58 (83%)
Disease duration (years)	12.2 (9.3)	14.6 (10.8)	14.1 (10.1)
RF or ACPA positive	21 (58%)	28 (82%)	49 (70%)
Current smokers	2 (6%)	8 (24%)	10 (14%)
BMI	26.2 (4.7)	26.9 (4.9)	26.5 (4.7)
No. with university‐level education	12 (33%)	11 (32%)	23 (33%)
***Comorbidities:***			
IHD	1 (3%)	5 (15%)	6 (9%)
Hypertension	12 (33%)	17 (50%)	29 (41%)
Diabetes	0	2 (6%)	2 (3%)
COPD	1 (3%)	3 (9%)	4 (6%)
Previous malignancy	2 (6%)	4 (12%)	6 (9%)
Previous csDMARDs	1.7 (0.9)	1.6 (0.9)	1.7 (0.9)
Previous bDMARDs	0.7 (1.4)	0.8 (1.2)	0.7 (1.3)
***Present medication:***			
csDMARD	25 (70)	30 (88)	55 (79)
MTX	24 (67)	29 (85)	53 (76)
MTX dose (mg)	18.9 (4.7)	18.7 (5.3)	18.8 (5.0)
csDMARD, combinations	7 (20)	3 (9)	10 (14)
bDMARD	3 (10)	10 (29)	13 (19)
TNFi	3 (8)	10 (29)	13 (19)
bDMARD, other	0 (0)	0 (0)	0 (0)
Prednisolone	7 (20)	5 (15)	12 (17)
Prednisolone dose (mg)	6.8 (2.4)	5.5 (2.7)	6.3 (2.5)
***Outcome variables:***			
DAS28	4.9 (0.9)	4.9 (0.9)	4.9 (0.9)
SJC	5.5 (3.2)	5.2 (3,3)	5.3 (3.3)
TJC	8.9 (5.5)	8.7 (5.1)	8.8 (5.3)
HAQ	1.1 (0.6)	1.2 (0.6)	1.1 (0.6)
RAID	4.9 (2.3)	5.6 (1.7)	5.2 (2.0)
PASS acceptable (%)	36 (13/36)	35 (12/34)	36 (25/70)
BMQ‐necessity	19.1 (4.3)	19.1 (3.2)	19.1 (3.8)
BMQ‐concerns	14.6 (4.8)	14.1 (3.8)	14.3 (4.3)
Patient satisfaction	8.4 (1.9)	8.3 (1.7)	8.4 (1.8)
EQ‐5D	0.45 (0.34)	0.47 (0.33)	0.46 (0.34)

ACPA: anti–citrullinated protein antibodies; bDMARD: biologic disease‐modifying anti‐rheumatic drug; BMI: body mass index; BMQ: Beliefs about Medicines Questionnaire (divided into necessity and concerns); COPD: chronic obstructive pulmonary disease; csDMARD: conventional synthetic disease‐modifying anti‐rheumatic drug; DAS28: Disease Activity Score in 28 joints; EQ‐5D: EuroQol‐5D; HAQ: Health Assessment Questionnaire; IHD: Ischaemic heart disease; MTX: methotrexate; PASS: Patient Acceptable Symptom State; RAID: rheumatoid arthritis impact of disease; RF: rheumatoid factor; SJC: swollen joint count; TJC: tender joint count; TNFi: tumour necrosis factor inhibitor.

The mean values for DAS28 and swollen joint count at baseline indicated that the patients had, on average, a disease activity well above the requirements according to the inclusion criteria.

### 3.1 Primary endpoint and secondary endpoints related to disease activity at week 26

In the PP analyses, the primary outcome (i.e., the difference in delta‐DAS28 between the IG and CG) was not statistically significant (0.43; 95% CI −0.27, 1.13) (Table 2).

**Table 2:** Changes within, and differences between, study groups at week 26 for primary and secondary outcomes (based on DAS28) when analysed per protocol

Primary outcome of disease activity (DAS28) at week 26				
	Baseline	Week 26	Differences at w26	
	Mean (SD)	Mean (SD)	Mean (95% CI)
Intervention group (*n* = 29)	4.67 (0.73)	3.17 (1.21)	1.50 (1.00, 2.00)	
Control group (*n* = 33)	4.94 (0.94)	3.87 (1.32)	1.07 (0.56, 1.57)	
*Group difference (95% CI)*				0.43 (−0.27, 1.13)

Secondary outcome of disease activity at week 26				
		***EULAR moderate/good response at week 26***	**Group difference (95% CI)**	
EULAR moderate/good response	Intervention group (*n* = 29)	76% (22/29)	27% (2, 49)	
	Control group (*n* = 33)	49% (16/33)		
		***DAS28 – Minimal clinical important improvement at week 26***		
DAS28 – Minimal clinically important improvement (DAS28 change of >0.6)	Intervention group (*n* = 29)	76% (22/29)	25% (1, 45)	
	Control group (*n* = 33)	51% (17/33)		
		***EULAR – low disease activity (DAS28 <3.2) at week 26***		
EULAR – low disease activity (DAS28 <3.2)	Intervention group (*n* = 29)	48% (14/29)	24% (0, 45)	
	Control group (*n* = 33)	24% (8/33)		

CI: confidence interval; DAS28: Disease Activity Score in 28 joints; EULAR: European League Against Rheumatology; SD: standard deviation.

In both the IG and CG, delta‐DAS28 improved significantly, by 1.50 (95% CI 1.00, 2.00) in the IG and 1.07 (95% CI 0.56, 1.57) in the CG.

A EULAR moderate or good response, which was a secondary outcome, was achieved by 76% (95% CI 58, 89) in the IG and 49% (95% CI 32, 65) in the CG, corresponding to a difference of 27% (95% CI 2, 49), in favour of the intervention.

At week 26, numerically larger improvements were also seen in the IG compared with the CG for other secondary outcomes based on DAS28, such as achieving low disease activity (IG: 48%, CG: 24%; difference: 24% [95% CI 0,45]) and having a minimal clinically significant improvement of >0.6 intervention (IG: 76%, CG: 51%; difference: 25% [95% CI 1, 45]) (Table 2).

In the ITT analyses, with LOCF, differences between the groups were in the same direction overall but slightly smaller in magnitude than in the PP analyses (Supporting Information Table S1).

### 3.2 Other secondary endpoints

In the PP analyses, there were no major differences between the groups in the improvement in any of the secondary outcomes based on patient‐reported information.

In both the IG and CG, there were significant improvements over 26 weeks in: RAID (IG: –1.31 [95% CI −2.31, –0.31]; CG: –1.17 [95% CI −2.12, –0.22]); PASS (IG: 28% [95% CI 5, 50]; CG 27% [95% CI 5, 50]); and patient satisfaction (IG: 1.00 [95% CI 0.18, 1.82]; CG: 0.55 [95% CI 0.04, 2.21]), but with no major difference between the two groups (Table 3).

**Table 3:** Changes within, and differences between, study groups at week 26 for secondary outcomes not reflecting disease activity when analysed per protocol

		Baseline values	Change at week 26 vs. baseline (95% CI)	
HAQ (range 0–3, best to worse)	Intervention group (*n* = 29)	1.1 (0.6)	−0.15 (−0.35, 0.05)	
	Control group (*n* = 33)	1.1 (0.6)	−0.42 (−0.67, 0.16)	
*Group difference (95% CI)*				−0.26 (−0.59, 0.06)
RAID (range 0–10, best to worse)	Intervention group (*n* = 29)	4.7 (2.2)	−1.31 (−2.31, −0.31)	
	Control group (N = 33)	5.5 (1.7)	−1.17 (−2.12, −0.22)	
*Group difference (95% CI)*				0.14 (−1.21, 1.49)
PASS (yes, %)	Intervention group (*N* = 29)	41% (12/29)	28% (5, 50)	
	Control group (*n* = 33)	33% (11/33)	27% (5, 50)	
*Group difference (95% CI)*				0 (−31, 31)
BMQ‐necessity (range 5–25, with 25 being the highest level of necessity)	Intervention group (*n* = 29)	18.7 (4.3)	0.68 (−1.04, 2.40)	
	Control group (*n* = 33)	19.3 (3.2)	0.26 (−1.39, 1.96)	
*Group difference (95% CI)*				0.39 (−2.73, 1.95)
BMQ‐concerns (range 5–25, with 25 being the highest level of concerns)	Intervention group (*n* = 29)	13.9 (4.8)	−1.14 (−2.82, 0.54)	
	Control group (*n* = 33)	14.3 (3.37)	−0.71 (−1.78, 0.36)	
*Group difference (95% CI)*				−0.43 (−1.48, 2.34)
Patient satisfaction (range 0–10, worst to best)	Intervention group (*n* = 29)	8.3 (1.9)	1.00 (0.18, 1.82)	
	Control group (*n* = 33)	8.3 (1.8)	0.55 (0.04, 2.21)	
*Group difference (95% CI)*				0.45 (−1.37, 0.46)
EQ‐5D (−1 to +1 (best health)	Intervention group (*n* = 29)	0.50 (0.32)	0.05 ([−0.12] – 0.21)	
	Control group (*n* = 33)	0.48 (0.33)	0.12 ([−0.04] – 0.28)	
*Group difference (95% CI)*				−0.08 (−0.15, 0.30)

BMQ: Beliefs about Medicines Questionnaire; CI: confidence interval; EQ‐5D: EuroQol‐5D; HAQ: Health assessment questionnaire; PASS: patient acceptable symptom state, RAID: Rheumatoid arthritis impact of disease.

### 3.3 Treatment from baseline to week 26

The proportion of patients given a bDMARD increased at week 26 vs. baseline in the IG (38% vs. 7%), as expected, due to the design of the intervention. A similar increase was also seen in the CG (46% vs. 27%), but at week 26 the proportion receiving a bDMARD was still smaller in the IG than in the CG.

A minority of patients received a 3‐week oral course of prednisolone (IG: 6/29; CG: 3/33) or an intra‐articular glucocorticoid injection (IG: 5/29; CG: 3/33), as expected, with a tendency for more patients in the IG than the CG to do so.

The proportions receiving a csDMARD (mainly methotrexate) increased modestly in both the IG and CG at week 26, as did the weekly dose of methotrexate (Table 4).

**Table 4:** Pharmacological treatments given to patients at baseline and at week 26, and intra‐articular glucocorticoid injections and doses given between weeks 0 and 26, by study group

	BASELINE		Week 26	
	Intervention group (*n* = 29)	Control group (*n* = 33)	Intervention group (*n* = 29)	Control group (*n* = 33)
csDMARD *n* (%)	23 (79)	29 (88)	24 (83)	30 (91)
MTX *n* (%)	23 (79)	28 (85)	24 (83)	30 (91)
MTX dose (mg)	18.9 (4.7)	18.7 (5.3)	19.0 (4.6)	19.5 (4.8)
csDMARD, combinations *n* (%)	6 (21)	3 (9)	6 (21)	4 (12)
bDMARD *n* (%)	2 (7)	9 (27)	11 (38)	15 (46)
TNFi *n* (%)	2 (7)	9 (27)	10 (35)	11 (33)
bDMARD, other *n* (%)	0	0	2 (7)	5 (15)
Prednisolone *n* (%)	5 (17)	5 (15)	4 (14)	4 (12)
Prednisolone dose (mg)	6.8 (2.4)	5.5 (2.7)	7.0 (2.7)	4.4 (1.3)
Weeks 0–26				
Intra‐articular glucocorticoids:				
No. of injections (*n*/patients)	NA	NA	8/6	6/3
Oral prednisolone courses (*n*/patients)			7/6	4/4
DMARD therapy changes:				
Changes in csDMARD therapy (*n*/patients)	NA	NA	5/5	5/5
Changes in bDMARD therapy (*n*/patients)	NA	NA	13/13	9/9

bDMARD: biologic disease‐modifying anti‐rheumatic drug; csDMARD: conventional synthetic disease‐modifying anti‐rheumatic drug; MTX: methotrexate; NA: not applicable; TNFi: tumour necrosis factor inhibitor.

### 3.4 Changes in disease activity and medication during follow‐up: Baseline to week 50

In the PP analyses, during the extended follow‐up from weeks 26 to 50, the extent of changes made with regard to csDMARD and bDMARD therapy were overall of similar magnitude in the IG and CG but occurred in both groups with lower frequencies compared with the initial study period (Supporting Information Table S2). However, eight patients in the CG received intra‐articular glucocorticoid injections (15 injections overall) compared with none in the IG (Supporting Information Table S2).

In both the IG and CG, delta‐DAS28 continued to improve, resulting in an overall improvement at week 50 compared with baseline, by 1.52 (95% CI 1.07, 1.97) in the IG and 1.33 (95% CI 0.72, 1.93) in the CG, with a mean difference at week 50 of 0.19 (95% CI −0.55, 0.93). During the same period of follow‐up, numerically larger, but statistically not significant, improvements were seen in the IG compared with the CG for: a EULAR moderate or good response (IG: 76%; CG: 60%), achieving low disease activity (IG: 48%; CG: 43%) and a minimal clinically significant improvement of >0.6 (IG: 31%; CG: 23%).

### 3.5 Reasons for discontinuation (adverse events)

A slightly larger number of patients withdrew from the study in the IG arm compared with the CG arm (seven vs. one) at week 26. Reasons for withdrawal in the IG were: lack of time and difficulties in attending scheduled appointments (*n* = 6) and a diagnosis of cancer (*n* = 1). The reason for withdrawal in the CG group was a diagnosis of cancer (*n* = 1). No patients withdrew owing to adverse effects of medication.

## 4 DISCUSSION

In the present study, inclusion was terminated prematurely, so we could not demonstrate significant superiority for a nurse‐led clinic built on principles of person centred care, tight control and a treat‐to‐target strategy compared with care as usual in patients with RA and moderate to high disease activity. Although the primary outcome, delta‐DAS28 at week 26, was not met, we did note the intervention to have significant effects for other outcomes based on DAS28, suggesting a benefit of the intervention strategy.

The numerically higher rates for changes made in csDMARD and bDMARD therapy at week 26 in the IG illustrated that the study strategy was followed. At week 50, differences in DAS28 still favoured the IG compared with the CG, but were numerically smaller than those seen at week 26. Possible explanations for these findings are general phenomena, such as regression to the mean or differences in treatment, with more intra‐articular injections being given in the CG than the IG during the extended follow‐up (Supporting Information Table S2).

In a recent review of seven randomized controlled trials (RCTs) (all but one based on patients with low disease activity or remission), it was shown that both disease activity and patient satisfaction improved more in the nurse‐led than the regular care groups after 2 years, although there was no difference between groups after 1 year (de Thurah, Esbensen, Roelsgaard, Frandsen, & Primdahl, 2017). The only previous study (Ndosi et al., 2014) which also included patients with moderate or high disease activity (mean DAS28 3.89 and 3.65 in nurse‐led and regular groups, respectively) showed a numerically but nonsignificantly larger improvement in the nurse‐led group, results that were in line with those of the present study.

Most previous studies have focused on patients with low disease activity, and often with a non‐inferiority design—that is, seeking to prove that nurse‐led clinics are “as good as” regular care with regard to safety and retaining a low disease activity level (Larsson et al., 2014; Ndosi et al., 2014; Primdahl et al., 2012). In the present study, the hypothesis was that nurse‐led care would have a larger effect than regular care in patients with moderate or high disease activity. Furthermore, the combination of optimizing the treatment with the goal of remission and by using person‐centred care is a unique approach compared with previous RCTs of nurse‐led clinics.

Patients from the IG also participated in a qualitative study after continuation of the present study, assessing their experiences with the provided care (Sjö & Bergsten, 2018). In these analyses, we showed that the patients experienced the intervention as “meeting competence”, with a “sustainable relationship” and that “they were doing a personal journey”. This is in line with earlier studies on patients' experiences of nurse‐led clinics (Arvidsson et al., 2006; Bala et al., 2012; Larsson et al., 2012).

As hypothesized, disease activity improved over the study period in the nurse‐led intervention. There was, however, a parallel and significant improvement in the regular care group, illustrating the difficulty of performing a comparative study with as complex an intervention as this in a regular care setting. Apart from the effect of regular care in itself, the effect in the CG may also reflect that the standard of care improved over the 3‐year study duration, due to both quality improvement work and increased resource allocations. Furthermore, according to the protocol, and for ethical reasons, patients in the regular care arm were offered an appointment with a physician at inclusion; this was taken up by 70% of patients and often led to change, or escalation in dose, of DMARD medication. This intervention could partly explain the improvements seen in the CG. It may also be the case that an outcome from the patient perspective, as shown in the questionnaire on RAID, with a focus on disease impact rather than disease activity, would have been a more realistic outcome of a study such as this, based on person‐centred care (Ferreira et al., 2018). Nevertheless, our results indicate that a nurse‐led clinic with our type of intervention may improve patient outcome in those with substantial disease activity. Furthermore, other authors have shown that a nurse‐led clinic is cost‐effective, in regard to DAS28 (Ndosi et al., 2014).

There were some limitations to the study. Firstly, we did not include the number of patients planned according to the power analyses, and this is likely to have prevented us from detecting statistically significant differences between groups. Nevertheless, we noted a tendency for larger improvements in our primary and secondary outcomes for disease activity in the IG compared with the CG. Secondly, there was a slight imbalance between some baseline mean values for the secondary outcomes, probably also due to the limited sample size, which hampered comparisons between groups for some of these outcomes. Thirdly, the fact that we assessed patients in the CG systematically at weeks 26 and 50 may, for some patients, have increased their awareness of disease activity, possible resulting in treatment changes. Fourthly, although nurses underwent structured training in person‐centred care, more extensive training might have led to a more effective intervention. Finally, during the 3‐year period over which the study was conducted, regular care was improved at the clinic.

The major strength of the study was that it was the first RCT to evaluate a clinic with a nurse‐led design, in RA patients with moderate or high disease activity only, and our results indicated that this strategy may have positive effects on disease activity. This strategy needs to be both further developed and evaluated in future studies, and also in other settings, where adaptations to local health care organization requirements may have to be done. Nevertheless, our results indicated that a nurse‐led clinic, according to the principles of the present study, may be a way forward to improve patient values and outcomes.

## FUNDING INFORMATION

This study was funded by the Swedish Rheumatism Association and University of Gothenburg Centre for Person‐centred Care (GPCC).

## CONFLICTS OF INTERESTS

The authors declare that there are no conflicts of interest.

## Supporting information

Table S1
Changes within and differences between study groups at week 26 for primary and secondary outcomes (based on DAS28) when analysed according to ITT with LOCF at week 26.
Table S2
Pharmacological treatments by treatment group, given to patients at baseline, week 26 and week 50 and for intra‐articular glucocorticosteroids injections and doses given during the randomized part of the study (week 0–‐26) and during the open follow‐up (week 26–‐50).
