# Supplementary material for: A randomized study comparing regular care with a nurse‐led clinic based on tight disease activity control and person‐centred care in patients with rheumatoid arthritis with moderate/high disease activity: A 6‐month evaluation
Source: Musculoskeletal Care. 2019 Jun 20;17(3):215–25. doi: 10.1002/msc.1403 (PMC6852576; doi:10.1002/msc.1403)
Supplement: Supplementary file 1 — Table S1 Changes within and differences between study groups at week 26 for primary and secondary outcomes (based on DAS28) when analysed according to ITT with LOCF at week 26. Table S2 Pharmacological treatments by treatment group, given to patients at baseline, week 26 and week 50 and for intra‐articular glucocorticosteroids injections and doses given during the randomized part of the study (week 0–‐26) and during the open follow‐up (week 26–‐50). [file MSC-17-215-s001.docx]

Suppl Table 1

Changes within and differences between study groups at week 26 for primary and secondary outcomes (based on DAS28) when analysed according to ITT with LOCF at week 26.

| Primary outcome of disease activity (DAS 28) at week 26 | | | | | | | | | | |
| --- | --- | --- | --- | --- | --- | --- | --- | --- | --- | --- |
|  | | Baseline | | Week 26 | Differences at w26 | | |  | | |
|  | | Mean (std) | | Mean (std) | Mean (95% CI) | | |  | | |
| IG (n= 36) | | 4.91(0.90) | | 3.52 (1.43) | 1.39 (0.97 – 1.82) | | |  | | |
| CG (n= 34) | | 4.92 (0.93) | | 3.89 (1.30) | 1.04 (0.54 – 1.53) | | |  |  |  |
|  |  |  |  |  | |  |  | |  |  |
| Group difference (95% CI) | |  |  |  | |  |  | 0.35  (-0.28 – 0.99) | | |

| Secondary outcome of disease activity at week 26 | | | |
| --- | --- | --- | --- |
|  |  | ***EULAR moderate/good response***  ***at week 26*** | **Group difference**  **(95% CI)** |
| EULAR moderate/good  response | IG (n=36)  95% CI:  CG (n=34) | 69% (25/36)  (53-82)  47% (16/34) | 22% (-1- 42) |
|  | 95% CI: | (31-64) |  |
|  |  | ***DAS 28 – Minimal clinical important***  ***improvement at week 26*** |  |
| DAS 28 – Minimal clinical important  improvement (DAS 28 – 0.6) | IG (n=36)  95% CI:  CG (n=34)  95% CI: | 69% (25/36)  (53-83)  50% (17/34)  (34-66) | 19% (-4 - 39) |
|  |  |  |  |
|  |  | ***EULAR – low disease activity (das 28<3.2) at week 26*** |  |
| EULAR – low disease activity  (das 28<3.2) | IG (n=36)  95% CI:  CG (n= 34)  95% CI: | 42% (15/36)  (27-58)  24% (8/34)  (12-40) | 18% (-4- 38) |
|  |  |  |  |

Suppl Table 2

Pharmacological treatments by treatment group, given to patients at baseline, week 26 and week 50 and for intra-articular glucocorticosteroids injections and doses given during the randomized part of the study ( week 0 -26) and during the open follow-up (week 26-50).

|  | BASELINE | |  | Week 26 | |  | Week 50 | |
| --- | --- | --- | --- | --- | --- | --- | --- | --- |
|  | IG (N=29) | CG (N=33) |  | IG (N=29) | CG (N=33) |  | IG (N=29) | CG  (N=33) |
| csDMARD (no (%)) | 23 (79) | 29 (88) |  | 24 (83) | 30 (91) |  | 24 (83) | 30 (91) |
| MTX (no (%)) | 23 (79) | 28 (85) |  | 24 (83) | 30 (91) |  | 24 (83) | 30 (91) |
| Mtx dose (mg) | 18.9 (4.7) | 18.7 (5.3) |  | 19.0 (4.6) | 19.5 (4.8) |  | 18.8 (4.8) | 19.7 (4.7) |
| csDMARD, other |  |  |  |  |  |  |  |  |
| csDMARD, combinations | 6 (21) | 3 (9) |  | 6 (21) | 4 (12) |  | 5 (17) | 6 (18) |
|  |  |  |  |  |  |  |  |  |
| bDMARD | 2 (7) | 9 (27) |  | 11 (38) | 15 (46) |  | 10 (34) | 16 (48) |
| TNFi | 2 (7) | 9 (27) |  | 10 (35) | 11 (33) |  | 8 (28) | 12 (36) |
| bDMARD, other | 0 | 0 |  | 2 (7) | 5 (15) |  | 2 (7) | 4 (12) |
|  |  |  |  |  |  |  |  |  |
| Prednsiolon | 5 (17) | 5 (15) |  | 4 (14) | 4 (12) |  | 3 (10) | 4 (12) |
| Prednisolon dose | 6.8 (2.4) | 5.5 (2.7) |  | 7.0 (2.7) | 4.4 (1.3) |  | 8.3 (2.9) | 5.0 (0) |
|  |  |  |  |  |  |  |  |  |
|  |  |  |  |  |  |  |  |  |
|  |  |  |  | Week 0 - 26 | |  | Week 26-50 | |
| Glucocorticsteroids i.a.: |  |  |  |  |  |  |  |  |
| No of injections (no/patients) | NA | NA |  | 8/6 | 6/3 |  | 0/0 | 15/5 |
|  |  |  |  |  |  |  |  |  |
| Oral Prednisolon courses (no/patients) | NA | NA |  | 7/6 | 4/4 |  | 5/5 | 3/3 |
| DMARD therapy changes: |  |  |  |  |  |  |  |  |
| Changes in csDMARD therapy (no/patients) | NA | NA |  | 5/5 | 5/5 |  | 1/1 | 1/1 |
| Changes in bDMARD therapy (no/patients) | NA | NA |  | 13/13 | 9/9 |  | 5/5 | 6/6 |
